# Transanal Minimally Invasive Surgery for Marsupialization of Chronic Abscess Cavity After Colorectal Anastomosis

**DOI:** 10.7759/cureus.38471

**Published:** 2023-05-03

**Authors:** Kevin R McMahon, Truong Ma

**Affiliations:** 1 Surgery, Summa Health, Akron, USA

**Keywords:** transanal minimally invasive surgery, tamis, coloanal, colorectal, marsupialization, anastomotic leak, low anterior resection

## Abstract

Anastomotic leaks are one of the most feared and morbid complications after colorectal anastomosis. Management of leaks depends on the severity of the leak and focuses on controlling sepsis and saving the anastomosis. The lower the anastomosis, the more amenable it is to transanal approaches for salvage. However, when a complication exists higher up in the rectum, the surgeon is more limited in the ability to visualize and intervene. With the advent of transanal minimally invasive surgery (TAMIS) and the advancement of endoscopic procedures, there are now more options for surgeons to visualize and intervene in anastomotic colorectal leaks. Prior reports have described the use of TAMIS for the management of anastomotic leaks in the acute phase. However, this same approach can be useful in the management of chronic leaks. This report highlights the benefit of TAMIS to allow visualization and marsupialization of a chronic abscess cavity following an anastomotic leak.

## Introduction

Anastomotic leaks are one of the most feared and morbid complications after colorectal anastomosis. The leak rate of colorectal anastomosis varies among studies but is generally in the range of 4% to 20% [1-5]. Management of leaks depends on several factors, including severity, patient's clinical stability, whether the leak is contained, whether the patient is proximally diverted, and the intraperitoneal versus extraperitoneal location of anastomosis. While considering these factors, the main principles focus on controlling sepsis and saving the anastomosis. Options are broad and range from percutaneous drainage to more extreme interventions such as re-anastomosis or colostomy. The lower the anastomosis, the more amenable it is to transanal approaches for salvage. However, when a complication exists higher up in the rectum, the surgeon is more limited in the ability to visualize and intervene. Recently, there has been an increase in options for surgeons to visualize and intervene in anastomotic colorectal leaks. These include endoscopic approaches as well as transanal minimally invasive surgery (TAMIS) [6-8]. Research of these techniques for anastomotic salvage has been limited to case reports and small series. These reports have focused on the management of anastomotic leaks in the acute phase. However, this same approach can be useful in the management of chronic leaks. This report highlights the benefit of TAMIS to allow visualization and marsupialization of a chronic abscess cavity following an anastomotic leak.

## Case presentation

Our patient was an 87-year-old female who underwent a colonoscopy due to symptoms of constipation and bleeding. Colonoscopy showed a large rectal mass, approximately 6 cm from the anal verge. Biopsies were taken, which showed well-differentiated adenocarcinoma. The complete staging was done. CT of the chest, abdomen, and pelvis was negative other than the known mass. Rectal MRI showed a rectal mass invading through the muscularis propria with two suspicious lymph nodes in the mesorectal fascia and a clear circumferential resection margin. The patient was classified with T_3_N_1_M_0_ disease. She was seen by medical oncology and was offered neoadjuvant chemoradiation. The patient declined; therefore, she was taken to surgery.

A robotic low anterior resection with coloanal anastomosis and diverting loop ileostomy was performed. Anastomosis was approximately 4 cm from the anal verge. The patient tolerated the procedure well and was discharged on postoperative day five without any issues. The pathology showed a 3 cm x 2.5 cm rectal mass that was removed with negative margins along with 17 lymph nodes, all negative for malignancy, giving her a T_2_N_0_M_0_ pathologic stage.

**Figure 1:**
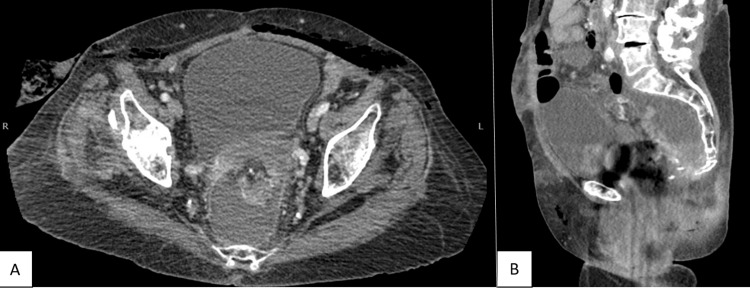
CT scan of the abdomen and pelvis two weeks after surgery. There was a fluid collection, representing an abscess cavity, posterior to the anastomosis. (A) Axial view showing the width of the collection. (B) Sagittal view showing the proximal extent of the collection.

At her follow-up visit two weeks after surgery, she was complaining of purulent drainage from her anus. CT scan was completed (Figure 1), which showed an abscess cavity posterior to the anastomosis, likely signifying an anastomotic leak (Grade B). A percutaneous drain was placed via a transgluteal approach for drainage and she was treated with antibiotics. The drain was removed 10 days later, after multiple days of no output. The patient did well until she presented two months later with rectal pain and drainage. CT scan was completed, which showed a persistent posterior abscess cavity.

**Figure 2:**
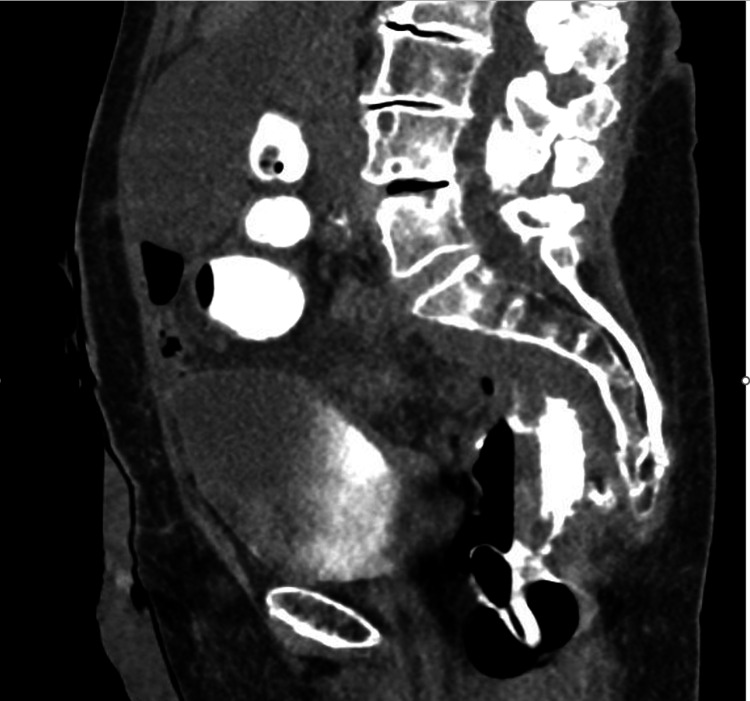
CT scan of the abdomen and pelvis with rectal contrast four months after surgery to evaluate for reversal. There is a persistent abscess cavity posteriorly seen with a small tract connected to the rectum. The tract and cavity were filled with contrast.

She was taken to the operating room for an exam under anesthesia with marsupialization of the abscess cavity using a laparoscopic stapler. Again, she did well until two months and then began to have purulent drainage from her anus with rectal pain. A CT scan with rectal contrast was completed, which showed that the patient had a persistent abscess cavity that now extended superiorly off the prior abscess cavity (Figure 2).

**Figure 3:**
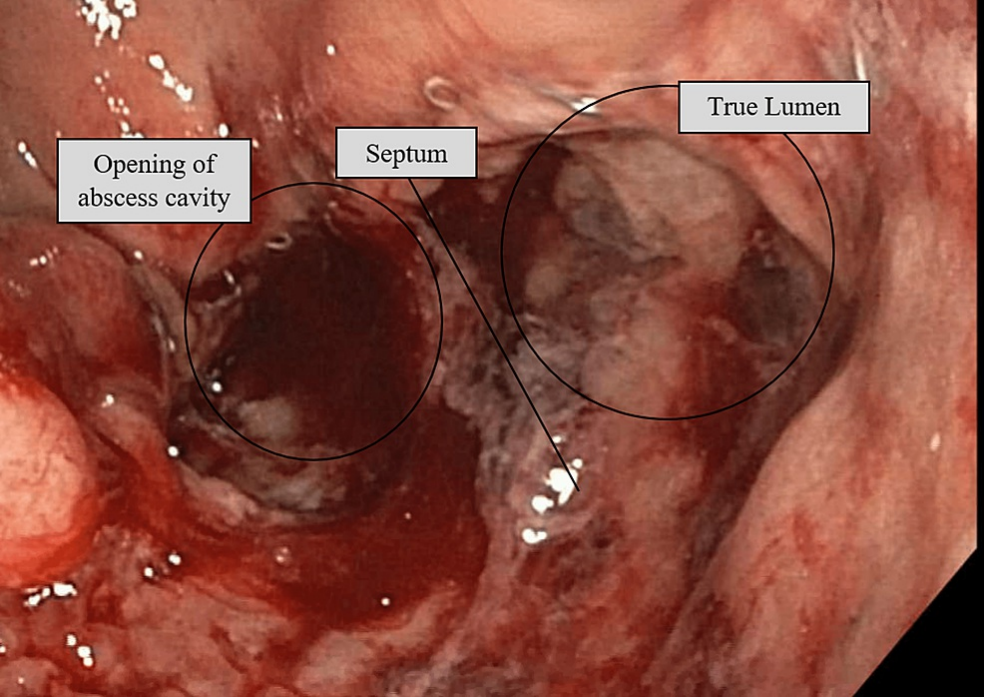
Endoscopic picture of the true lumen and opening of the abscess cavity prior to septotomy. The opening of the abscess cavity was very small, which is likely why the abscess did not drain completely and recurred.

**Figure 4:**
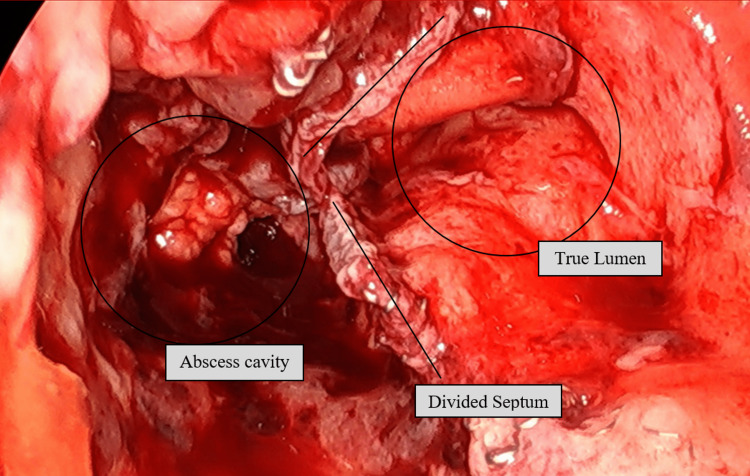
Laparoscopic image through the TAMIS port of the abscess cavity and colon lumen after septotomy completed with the laparoscopic stapler. This is the view of the colon lumen through the TAMIS port, which allows visualization using the laparoscope while also being able to work with the laparoscopic instruments. The abscess cavity has now been marsupialized and is completely open to the colon lumen to allow complete drainage. TAMIS: transanal minimally invasive surgery.

She was taken again for a rectal exam under anesthesia with a plan for repeat marsupialization. On exam, the abscess cavity could not be directly palpated. Flexible sigmoidoscopy was used to examine the area, which was approximately 8 cm from the anal verge. The true lumen was identified, but the opening to the abscess cavity was very small, and nearly undetectable (Figure 3). The scope was used to attempt to enter the abscess cavity, but it could not be passed. Manual palpation of the area was attempted, but it was impossible to visualize and difficult to palpate adequately. Given the difficulty, a TAMIS port was placed and the colon was insufflated. This allowed much-improved visualization of the area and allowed for the simultaneous use of our instruments. Electrocautery was used to enlarge the opening of the abscess cavity, once enlarged, a laparoscopic stapler was used to perform a septotomy and marsupialize the abscess cavity. Two 45 mm loads were used. After stapling, the abscess cavity was contiguous with the true lumen of the colon (Figure 4). The patient was extubated without issue and discharged home. She was seen in follow-up and has not had any recurrence of symptoms for over one year. Throughout all of this, she has done well with her ileostomy and has elected to keep the ileostomy rather than proceed with any type of evaluation for reversal.

## Discussion

Despite advances in rectal cancer treatment, anastomotic leaks occur in 4-20% of low anterior resections [1-5]. The management of these leaks depends on the severity of the leak and the clinical status of the patient. The goal in managing these leaks is to control sepsis and avoid permanent stoma creation.

Because anastomotic leak is such a major complication of colorectal surgery, the International Study Group of Rectal Cancer published a classification system for anastomotic leaks in 2010 [9]. Under this system, a grade A leak does not require any change in management. Grade B leaks require an intervention, but do not require re-operation. And grade C leaks require re-operation [9]. Under this classification, re-operation is necessary when a leak cannot be controlled with antibiotics and percutaneous drainage alone. Historically, re-operation in these situations usually results in proximal diversion or a Hartman’s procedure [10,11]. However, as technology has changed and there are more options available to surgeons, the equation is evolving. More recently, there have been reports of managing leaks using endoluminal wound vacuums and stents or repairing the defect using transanal approaches [7,8,12,13]. Although transanal approaches technically qualify as a need for re-operation, they allow the leak to be addressed with less morbidity than an intra-abdominal operation and stoma creation. Further research is needed to determine the success rates and long-term outcomes of such approaches to managing anastomotic leaks.

In this presented case, sepsis was prevented with percutaneous drain placement. Unfortunately, a chronic abscess cavity developed, and the patient continued to have symptoms. Management options for chronic pre-sacral abscess include proctectomy, marsupialization, or endoluminal wound vacuum [12,14]. Ideally, minimally invasive approaches should be attempted first to reduce morbidity, especially the need for a permanent stoma. Marsupialization has benefits over endoluminal wound vacuum given the problem can be solved with only one intervention versus multiple interventions required for vacuum exchanges. However, being able to marsupialize the cavity to the extent that all the contents can drain freely and there will not be a remaining dependent portion hinges on being able to completely visualize or palpate the area. TAMIS is a versatile tool that can be used to better visualize the rectum and intervene in pathology. The TAMIS approach can be used with standard laparoscopic instruments, or it can be combined with a robotic approach to allow for more freedom of movement in the tight space of the rectum. In our case, standard laparoscopic instruments were sufficient to marsupialize the cavity; however, if a more complex repair is required, such as suture repair, a robotic approach may be beneficial. Although there is no clear data examining this topic, extrapolation of data comparing the laparoscopic vs. robotic approach in the abdomen, which shows lower conversion rates to open surgery with the use of the robot, would indicate an increased likelihood of success when a complex repair is needed in a tight space [15].

In light of the increase in available technologies, there is no single best approach for the management of anastomotic leak after colorectal surgery. Management decisions should be tailored to each patient’s clinical presentation and anatomic findings to control infection and minimize morbidity.

## Conclusions

TAMIS is a useful tool that can extend a surgeon’s capabilities to visualize and address a variety of problems in the rectum. Our case highlights the benefit of utilizing TAMIS to improve visualization and completely marsupialize a chronic abscess cavity that would otherwise be too high and difficult to complete with a standard transanal approach. TAMIS serves as a useful tool for surgeons as they deal with anastomotic leaks in both acute and chronic settings.
